# A Case of Degenerated Bronchocele of Enormous Size, Cured by Medical and Surgical Treatment

**Published:** 1860-01

**Authors:** O. B. Knode

**Affiliations:** St. Joseph, Missouri


ART. II.—*A Case of Degenerated Bronchocele of enormous Size, cured by Medical and Surgical Treatment.* By O. B KNODE, M.D., of St. Joseph, Missouri.

JOSEPH REMELE, of Palermo, Kansas Territory, aged thirty-seven years, a native of Switzerland, but a resident of this country for nine years, presented himself to me for the first time about fourteen months ago, with an enormous tumor situated on the anterior part of the neck and breast. He stated to me that about fourteen years ago the tumor commenced to enlarge, and grew slowly until the last four or five years, when it increased more rapidly, during which time he suffered a good deal of pain and uneasiness, rendering him incapable of labor. When in his native country he had used some local applications to it, prescribed by a physician, but since his residence here had done nothing for it. The middle of the base of the tumor was situated about the region of thyroid gland, extending on either side nearly as far as the acrominal ends of the clavicle. The tumor measured eighteen inches in length, reaching some distance below the ensiform cartilage, and had a circumference of a little more than two feet; it hung pendulous, and detached from the chest about half its length, so that the hand could be pushed between it and the sternum and ribs, which were greatly depressed and thinned by absorption, produced by the long-continued pressure and great weight of the tumor. It fluctuated on percussion, but was so tense that nothing but fluid could be detected. Looking upon it as a case of degenerated bronchocele, and with the exploring-needle satisfying myself that it contained a fluid, I plunged a bistoury into its most pendulous point, and withdrew a gallon and three pints of inodorous and insipid fluid, about the color and consistence of weak coffee; after its withdrawal, the great tension being relieved, I could discover within the immense remaining wrinkled sac a large, contorted, nodulated, hard tumor, as large as two stout fists, which seemed to have two or more attachments, one seemingly to the hyoid bone and thyroid cartilage, as large apparently as the wrist. Another attachment, about half as large as the first, seemed to have its insertion about the origin of the left sterno-cleido-mastoid muscle, and another intermediate, which I could not well define. When the tumor was lifted up, and grasped firmly in the hand, the enlarged thyroid arteries could be felt pulsating. I determined to remove the tumor with the écraseur, provided nothing could be done by medicine and other means; yet I intended to give him the full benefit of the iodides, before resorting to so serious an operation. I consequently strapped the tumor as tightly as possible with adhesive plaster, and put him upon iodide of potassium, five grains three times a day. I did not see him again for two weeks, when he sent for me, and I found the straps had become loose, and the orifice of the wound made for the exit of the fluid had closed, and a reaccumulation of it, to the amount of half a gallon, had taken place, which I at once removed, leaving this time a tent in the wound, to be withdrawn every day or two, to permit the fluid to escape; a good deal of blood was mixed with the serum at this time. I found also that the large knobby internal tumor had somewhat diminished in size, and was much softer to the touch. I strapped it again tightly, and continued the same prescription, increasing each dose two grains. The wound continued to discharge for five or six days, when it entirely ceased, and the tumor commenced to decrease rapidly in size; and, under the use of the same prescription for two months longer, was almost entirely removed. To-day, October 20th, 1859, nearly fifteen months since I first saw him, he walked a distance of ten miles to visit me, in the enjoyment of most excellent health, and greatly delighted at the removal of the immense load he had been carrying for so many years. The site of the former tumor is now only perceptible in a flabby, wrinkled state of the skin, as large perhaps as the hand, with the thyroid gland as large as a small orange, and very hard, and he tells me that it is still growing gradually less; his health and spirits are excellent, and is once more enabled to do all sorts of work and in all kinds of weather; and, finally, what is always so agreeable to the physician, can scarcely find words sufficient, in his broken German, to express his great gratitude for his deliverance from his terrible burden.

